# Taking the bull by the horn: the frontline use of infliximab for the treatment of immune checkpoint inhibitor-induced enterocolitis

**DOI:** 10.1186/s40425-018-0480-1

**Published:** 2018-12-22

**Authors:** Khashayar Esfahani

**Affiliations:** Department of Medical Oncology, Segal Cancer Center, Jewish General Hospital, McGill University, Montreal, Canada

## Abstract

Immune checkpoint inhibitors which activate the host’s immune system to fight cancer have brought dramatic improvements to the overall survival of a growing number of deadly malignancies. Their use comes at the expense of often serious immune-related adverse events which consist of an off-target attack of the immune system on potentially any of the human body’s healthy organs. For lack of better-validated evidence, and regardless of the organ affected, clinicians often use the same immunosuppressive regimens consisting of high dose corticosteroids followed by the introduction of biologic agents such as the tumor-necrosis alpha inhibitor infliximab for corticosteroid-refractory toxicities. The article by Johnson et al. is timely in providing a more personalized approach for the management of immune-related toxicities affecting the lower digestive tract with many positive clinical outcomes associated with the upfront use of infliximab in association with corticosteroids. This commentary will provide a narrative summary of their findings in light of the current clinical knowledge relevant to the understanding of immune-related enterocolitis.

## Commentary

Immune-related enterocolitis (irEC) is a common side effect associated with the single use or the combination of cytotoxic T-lymphocyte antigen 4 (CTLA-4) or programmed death 1 (PD-1) immune checkpoint inhibitors (ICI) in various malignancies. Patients often present with painless watery diarrhea, sometimes severe enough to warrant hospitalization and intravenous corticosteroid administration. Since the early days following the introduction of these agents into clinical practice, significant ground has been gained in recognizing irEC as a serious complication of ICI which can lead to pancolitis, perforation, sepsis and ultimately the demise of the patients if not promptly recognized and aggressively treated [1].

Johnson et al. provide interesting data with regards to the clinical outcomes of patients experiencing iREC depending on the frontline immunosuppressive regimens used. In a single-institutional review, they compare the outcomes of 39 patients receiving corticosteroids alone vs. 36 patients receiving the upfront combination of the tumor necrosis factor (TNF) inhibitor infliximab plus corticosteroids. Despite a higher incidence of higher grade irEC in the infliximab plus corticosteroid group compared to those having received corticosteroids alone (86% vs. 34% grade 3/4 irEC), the median time to diarrhea resolution (3 vs. 9 days), the time to steroid dose de-escalation (4 vs. 13 days) and the overall corticosteroid exposure (35 vs. 51 days) all favored the infliximab arm. The current American Society of Clinical Oncologists (ASCO) treatment guidelines for irEC recommend the use of corticosteroids plus TNF inhibitors only in patients who are refractory to frontline corticosteroids and fail to achieve a significant clinical improvement of their diarrhea within 48 to 72 h of treatment initiation [2]. It is important to highlight that these guidelines are based mostly on expert opinion rather than on solid prospective clinical trial evidence. Johnson et al.’s data, despite being small in number, retrospective and single-institutional, challenge the status quo and provide a rationale for more aggressive upfront treatment of severe cases of irEC. Their findings are in line with other recently published evidence which seem to suggest that combination immunosuppressive therapy upfront for all patients with irEC will likely achieve superior clinical outcomes when compared to the use of corticosteroid therapy alone [3].

There is often a tendency to compare immune-related adverse events (irAE) to their classic autoimmune counterparts. For instance, in the case of irEC, a comparison is often drawn to inflammatory bowel diseases (IBD) such as Crohn’s and ulcerative colitis. Despite sharing many clinical similarities, histopathological correlations between these two disease entities are concordant in less than half of the patients [1]. As highlighted in the study of Johnson et al., 72% of patients had a clinical resolution of their symptoms after a single infusion of infliximab, with no patients requiring more than 3 doses of this treatment. This again underscores a critical difference between IBDs and irEC, the latter being likely the consequence of a transient immune activation which very rarely develops into a chronic autoimmune disease if aggressively treated. TNF inhibitors are nevertheless exquisitely sensitive in both settings to induce clinical and histopathological remissions.

Many cytokines, like the TNF alpha, often play a pleiotropic role in the setting of malignancy, on one hand promoting inflammation and driving immune side effects and, on the other hand, playing a crucial role in immune surveillance aimed at keeping the malignancy in check [4]. Like a Pandora’s box, one might not know what potential end-results to expect when biologic agents are thrown in the mix. As a comparable example, interleukin-17 (IL-17) is another pleiotropic cytokine that also plays a critical role in the pathology of immune colitis as well as in cancer immune surveillance. In support of this hypothesis, we previously reported a case of irEC and psoriasis successfully treated with an IL-17 antagonist but with the ultimate end-result of reversal of the anti-tumor efficacy from checkpoint inhibitors [5]. It is thus reassuring to see that the use infliximab in the study by Johnson et al. did not seem to impair immune surveillance, although translational and prospective studies of larger sample size are clearly needed to establish the dynamics of these biologic agents on cancer-related outcomes. It’s also important to highlight that infliximab is one of many TNF inhibitors available for clinical use, and others, such as adalimumab, have also been used in the setting of irEC with good efficacy and with the added convenience of using a subcutaneous route of administration which is often favored by patients [6]. Another emerging and promising biologic agent for the treatment of irEC is vedolizumab [7]. This humanized murine antibody against the α4β7-integrin on the surface of CD4+ T cells prevents binding to its ligand MAdCAM-1, which is expressed on the endothelial surface of the gut and its associated lymphoid tissues. As a result, lymphocytes are unable to bind and to extravasate into the gut mucosa, a key step in the pathogenesis of irEC. Despite its interesting gut-specific mode of action, vedolizumab should be used with caution in fulminant cases of irEC, as it has a much slower onset of action compared to TNF inhibitors [7]. Finally, a promising recent report demonstrated the efficacy of stool transplantation for two cases of irEC refractory to corticosteroids, infliximab, and vedolizumab [8].

Despite being very informative, a limitation from the study of Johnson et al. is the lack of clinical information on the subsequent anti-cancer treatments used for the patients in their retrospective cohort. Following the occurrence and the resolution of a grade 3–4 irAE, most clinicians favor clinical observation rather than re-challenging patients with further ICI for fear of potentially life-threatening recurrent autoimmune toxicities. Furthermore, clinical observation suggest that some patients seem to derive ongoing clinical benefit after a severe irAE, which is often used as a surrogate marker of successful immune activation, without being rechallenged with further ICI. According to ASCO’s clinical guidelines, severe grade 3–4 reactions are ground for permanent ICI discontinuation. However, in some diseases such as metastatic melanoma which accounted for the majority of the cases in Johnson et al.’s study, treatment options outside ICI are often limited. Clinicians are thus left with the dilemma of whether they should rechallenge a patient who was benefitting from ICI up to the point of a major irAE, and whose tumor starts to progress after a period of clinical observation following the resolution of all toxicities. Recent data suggest that patients experiencing grade ≥ 3 irEC on the CTLA-4 and PD-1 combination for the treatment of metastatic melanoma can safely resume PD-1 alone upon disease progression [9]. Further evidence suggests that the immunopathological features of CTLA-4-induced irEC are different than the ones that are PD-1-induced [10]. As such, one could theoretically safely re-challenge a patient having developed irEC on a CTLA-4 inhibitor with a PD-1 inhibitor upon disease progression, and vice-versa. If switching is not an option, one can also rechallenge with the same agent with the risk of developing the same irAE in over half of the patients [11]. Positive results from the study of Johnson et al. thus opens the possibility of studying ICI in combination with TNF inhibitors as a prophylactic method in patients at high risk of recurrent irEC in a rechallenge setting.

In summary, the study by Johnson et al. provides new clinical insights in the management of irEC which is likely to challenge the current treatment guidelines for the management of these toxicities. The findings from this study warrant to be further validated in larger prospective randomized clinical trials.
